# PKCδ Protects against Lupus Autoimmunity

**DOI:** 10.3390/biomedicines12061364

**Published:** 2024-06-19

**Authors:** Sailee Vijay Chavan, Shreya Desikan, Christopher A J Roman, Chongmin Huan

**Affiliations:** 1 Program in Molecular and Cellular Biology, The School of Graduate Studies, State University of New York (SUNY) Downstate Health Sciences University, Brooklyn, NY 11203, USA; sailee.chavan@downstate.edu (S.V.C.); shreya.desikan@downstate.edu (S.D.); 2 Department of Cell Biology, State University of New York (SUNY) Downstate Health Sciences University, Brooklyn, NY 11203, USA; christopher.roman@downstate.edu

**Keywords:** PKCδ, SMS2, lupus, autoimmunity, B cell tolerance

## Abstract

Protein kinase C delta (PKCδ) has emerged as a key protective molecule against systemic lupus erythematosus (SLE or lupus), an autoimmune disease characterized by anti-double stranded (ds) DNA IgGs. Although PKCδ-deficient mice and lupus patients with mutated *PRKCD* genes clearly demonstrate the requirement for PKCδ in preventing lupus autoimmunity, this critical tolerance mechanism remains poorly understood. We recently reported that PKCδ acts as a key regulator of B cell tolerance by selectively deleting anti-dsDNA B cells in the germinal center (GC). PKCδ’s tolerance function is activated by sphingomyelin synthase 2 (SMS2), a lipid enzyme whose expression is generally reduced in B cells from lupus patients. Moreover, pharmacologic strengthening of the SMS2/PKCδ tolerance pathway alleviated lupus pathogenesis in mice. Here, we review relevant publications in order to provide mechanistic insights into PKCδ’s tolerance activity and discuss the potential significance of therapeutically targeting PKCδ’s tolerance activity in the GC for selectively inhibiting lupus autoimmunity.

## 1. Lupus Remains a Significant Clinical Challenge

Lupus is a debilitating, chronic, and clinically heterogeneous autoimmune disease characterized by anti-double stranded (ds)-DNA IgGs, which contribute to autoimmune-complex-mediated systemic organ injury [1,2]. Lupus affects about 1.5 million Americans, 90% of whom are women; women of color are more frequently and more severely affected. Although female sex hormones are thought to facilitate the development of lupus autoimmunity, recent evidence points to the dosage of X chromosome genes as a key instigator of the female-bias of the disease. For example, male patients with Klinefelter’s syndrome bearing an extra X-chromosome (47, XXY) have a dramatically increased risk of developing lupus, while women with Turner’s syndrome (45, XO) have a reduced risk for lupus [3,4]. Other genetic, environmental, and immunological factors, and even some medications, have also been found to promote lupus autoimmunity. However, the interplay between these risk factors and their relationship to the fundamental defects essential for lupus pathogenesis remains poorly understood. This impedes the design of mechanism-oriented treatment approaches that can specifically and effectively suppress lupus autoimmunity but spare normal body functions. Indeed, current treatment options lack sufficient specificity and effectiveness, often suppressing protective immunity and causing other adverse effects [5,6]. As a result, lupus remains a severe clinical challenge, causing a significant social and economic burden [7,8,9].

The medications commonly used for lupus treatment include steroids, nonsteroidal anti-inflammatory drugs (NSAIDs), other general immunosuppressants, and disease-modifying anti-rheumatic drugs (DMARDs) such as antimalarial agents and biologics. One antimalarial agent, hydroxychloroquine (HCQ), has been used as the first-line treatment for lupus for nearly 60 years. Multiple large-scale studies have confirmed that, as an immunomodulator, HCQ can reduce lupus activity and improve patient outcomes over time without significantly increasing the risk of infection or malignancy [10]. Nevertheless, adverse side effects limit the use of HCQ; the long-term use of HCQ results in HCQ accumulation, particularly in patients with renal impairment, which may cause nausea, vomiting, diarrhea, and more severe complications, such as retinopathy and cardiotoxicity. These adverse events often force patients to reduce or even discontinue their HCQ treatment, resulting in lupus flares [10,11]. Despite HCQ’s long history as a first-line treatment for lupus, the mechanism of its action is not fully understood. Many studies have suggested that HCQ may work by moderately inhibiting the signaling of Toll-like receptors 7 and 9 (TLR7 and TLR9) in cells like plasmacytoid dendritic cells (pDCs) and B cells [12,13]. However, to what extent TLR7 and TLR9 participate in its effects, and in which cell type those effects are most critical, is still unresolved. For example, although HCQ suppressed lupus autoimmunity in lupus-prone MRL/lpr mice [14,15], IRS 954, a more potent inhibitor of both TLR7 and TLR9, could not suppress the production of anti-dsDNA antibodies in MRL/lpr mice [16]. In contrast, IRS 661, a TLR7-specific inhibitor, significantly reduced the production of anti-dsDNA antibodies [16], consistent with the role of the B cell TLR7 and TLR9 in promoting and inhibiting lupus autoimmunity respectively [17]. Taken together, these findings indicate that the inhibition of both TLR7 and TLR9 cannot fully explain the beneficial effects of HCQ in lupus and point to other as-yet unknown functions of HCQ, including its potential effects on other cells like IFNalpha-producing pDCs that are involved in lupus autoimmunity.

Steroids, NSAIDs, and other general immunosuppressants are also commonly used to relieve lupus pathogenesis. However, the therapeutic effects of these medications are achieved by the general suppression of immune and inflammatory responses, which unavoidably sacrifices normal immunity and increases the risk of infection. Furthermore, other adverse events, such as gastrointestinal irritation, hepatic toxicity, hypertension, weight gain, osteoporosis, and renal dysfunction are also frequently observed in lupus patients receiving these medications [5,6]. The non-specific action of these treatments and their many adverse effects make them an inadequate long-term treatment option for lupus, emphasizing the need for more specific treatments.

In addition to the above mentioned medications, the biologic belimumab, a neutralizing antibody against B cell-activating factor (BAFF), has been used as an adjunct to standard therapy for lupus based on evidence that BAFF plays a role in promoting lupus autoimmunity [18]. BAFF is an essential pro-survival cytokine required for B cell survival and for maintaining homeostasis [19]. More than half of lupus patients have upregulated BAFF in their circulation [20], and transgenic mice over-expressing BAFF exhibit a lupus-like phenotype [21]. Multiple clinical studies have demonstrated that belimumab is well tolerated and beneficial in patients with lupus but has modest efficacy [22,23,24,25,26,27,28]. These studies also showed that belimumab may significantly reduce total B cell numbers, with a 6% to 38% rate of adverse events, including infections, infusion reactions, and hypersensitivity reactions. Another antibody used for lupus treatment is rituximab. Rituximab non-selectively depletes B cells by binding to CD20 on the B cell surface, which diminishes normal B cell-mediated immune responses [29]. Therefore, while these biologics provide some targeting of cells that drive lupus disease, they still come with the consequence of hampering normal B cell immune function.

Collectively, the presented evidence underscores that there remains a lack of medications that selectively and effectively suppress lupus autoimmunity, limiting our ability to support patients with this disease. Addressing this serious clinical challenge will require a new therapeutic strategy that selectively targets the common and fundamental defects essential for the development of lupus autoimmunity.

## 2. Insufficient Germinal Center B Cell Tolerance Enables Lupus Autoimmunity

B cell tolerance serves as an essential self-defense mechanism against autoimmunity by deactivating or deleting autoreactive B cells that naturally arise in the body [30,31,32,33]. Autoreactive B cell receptors (BCRs) are generated by essential physiologic processes in B cell development: VDJ recombination in the bone marrow and somatic hypermutation (SHM) in the germinal center (GC). The random nature of these two genomic alterations radically diversifies the range of BCR specificities, and SHM also increases BCR affinity, which is required to develop a diverse anticipatory repertoire of antigen-binding specificities needed to effectively protect against a virtually limitless array of pathogens, but which also unavoidably generates some autoreactive BCRs. Although the exact mechanisms are not fully elucidated, the existence of B cell tolerance checkpoints in the bone marrow and periphery, including the GC, is well established. In the setting of autoimmunity, the breakdown of these B cell tolerance mechanisms allows for the survival of these spontaneously generated autoreactive B cells, which cause the development of disease [30,31,32,33].

The pathogenesis of lupus is mainly driven by anti-nuclear antibodies, most prominently anti-dsDNA IgGs [1]. Studies of autoreactive B cells in lupus patients and mouse lupus models have shown that reversing SHM in the BCR gene of anti-dsDNA B cells removes lupus autoimmunity in these cells, thus identifying SHM as the major generator of anti-dsDNA BCRs and highlighting the importance of GC B cell tolerance for preventing lupus autoimmunity [34,35,36,37,38,39]. Indeed, insufficient GC B cell tolerance has been recognized as the key defect that permits the survival of autoreactive B cells in the GC and their subsequent terminal differentiation into autoreactive plasma cells and memory B cells, contributing to the development of lupus disease [40,41]. Therefore, it appears that pursuing a treatment strategy that restores GC B cell tolerance in lupus patients would be a reasonable approach to address the issues of general immune suppression and off-target toxicities in the current lupus treatments, as it should ideally suppress lupus autoimmunity while sparing normal body functions. However, how GC B cell tolerance selectively deletes autoreactive B cells and how lupus patients lose the function of this essential self-protective mechanism in the GC are largely unknown. Therefore, further understanding of the mechanistic aspects of GC B cell tolerance is urgently needed to capitalize on this conceptual strategy, which is to treat lupus by strengthening GC B cell tolerance.

## 3. PKCδ Is Required to Prevent Lupus Pathogenesis

The protein kinase C (PKC) family is a group of highly related serine/threonine kinases that plays diverse regulatory roles in cell proliferation, differentiation, and apoptosis. By phosphorylating serine and threonine residues on a large number of proteins, PKC isozymes serve as cytoplasmic signal transducers that mediate cellular responses from the plasma membrane to the nucleus [42]. In addition, studies have shown that PKC isozymes can function as chromatin-associated kinases to regulate cellular activities [43]. Dysregulated PKC isozymes have been found to promote the development of various pathologies, such as heart diseases, dermatological diseases, psychiatric diseases, metabolic diseases and neurological diseases [44]. In the immune system, defective PKC isozymes may result in various autoimmune diseases including multiple sclerosis, inflammatory bowel disease, rheumatoid arthritis, Type I diabetes, celiac disease, and lupus [45]. Although numerous efforts have been made to therapeutically target PKC isozymes, none have been reported that can specifically target individual PKC isoforms for disease treatment [44]. This is an important shortfall to be addressed, as drugs that generally target the PKC family lack selectivity and result in many off-target effects due to the high degree of homology among the PKC isozymes. This lack of selectivity, coupled with the inadequate understanding of disease-specific roles of the PKC isozymes, present a large challenge in translating our knowledge of defective PKC isozymes in disease to effective treatments [44]. Despite all these challenges, targeting individual PKC isozymes remains an important potential approach for many unmet clinical needs.

The PKC family is subdivided into classical PKCs, novel PKCs, and atypical PKCs. Classical PKCs have binding sites for both diglycerol (DAG) and calcium ions (Ca^2+^), both of which are required to be bound for their activation. Novel PKCs only possess a binding domain for DAG, and binding of DAG alone is sufficient for activation. Atypical PKCs, on the other hand, lack binding domains for either DAG or Ca^2+^, and are activated through unknown mechanisms [42]. It is known that intracellular PKC isozymes can be activated by the phospholipase C (PLC) family in response to the binding of a variety of hormones, growth factors, and neurotransmitters to the corresponding cell surface receptors. Activated PLC hydrolyzes PtdIns(4,5)P_2_ into DAG and Ins(1,4,5)P_3_. Ins(1,4,5)P_3_ triggers the release of Ca^2+^ from the endoplasmic reticulum (ER), which, in turn, activates classical PKCs together with DAG. On the other hand, DAG derived from PtdIns(4,5)P_2_ may activate novel PKCs by itself. In principle, it is reasonable to propose that novel PKCs could therefore also be activated by other known DAG producers. For example, sphingomyelin synthase (SMS) was proposed to activate PKC three decades ago [46]. SMS produces sphingomyelin (SM) and DAG by the transfer of phosphocholine from phosphatidylcholine onto ceramide. SMS has two isoforms: SMS1, which primarily resides on the Golgi membrane, and SMS2, which is prominently located on the plasma membrane [47]. However, physiological evidence directly supporting the activation of PKCs by SMS1 or SMS2 had not been described until our research group recently reported that SMS2 specifically binds to and activates PKCδ, but not other PKCs, in B cells [48]. PKCδ is a novel PKC known to prevent lupus autoimmunity in both humans and mice [49,50,51,52,53,54,55,56,57,58]. Mutations in *PRKCD*, the gene encoding the human PKCδ protein, cause an autosomal-recessive form of juvenile-onset lupus (Table 1). Consistently, *Prkcd* knockout mice exhibit a lupus-like phenotype [57,58].

## 4. PKCδ Is Involved in B Cell Tolerance

In line with the requirement for PKCδ in preventing lupus autoimmunity, it has been shown that the nuclear translocation of PKCδ, a proapoptotic activity in B cells, is blocked by BAFF [61]. More specifically, spontaneous apoptosis in resting B cells is regulated by the nuclear localization of PKCδ that contributes to phosphorylation of histone H2B at serine 14 (S14-H2B), which is associated with cell death [62]. However, treatment with BAFF promotes the survival of B cells by preventing PKCδ nuclear translocation. Given that BAFF promotes the development of lupus autoimmunity, and PKCδ protects against lupus autoimmunity, this finding suggests the involvement of PKCδ nuclear translocation in B cell tolerance. In support of this view, PKCδ nuclear translocation in B cells was found to be defective in a mouse lupus model [63]. Notably, in both the studies mentioned above, PKCδ nuclear translocation was found to be induced in cultured B cells but not in untouched naïve B cells, suggesting that some type of metabolic stress induced by culturing was promoting the PKCδ nuclear localization. We reported that although naïve B cells contain much more cellular PKCδ than GC B cells, nuclear PKCδ was observed in GC B cells but was almost undetectable in naïve B cells [48], suggesting that in vivo, PKCδ nuclear translocation and its tolerance activity is unique to GC B cells.

Other studies have shown that PKCδ may regulate tolerance in bone marrow B cells and in peripheral B cells at the transitional stage via different mechanisms of B cell tolerance. Limnander et al. have showed that in immature bone marrow B cells, the Ca^2+^-dependent activation of ERK promotes antigen-induced apoptosis [59]. PKCδ and the guanine nucleotide-exchange factor RasGRP are required for the activation of this Ca^2+^-dependent tolerance pathway. In splenic transitional B cells, PKCδ is also essential for activating the proapoptotic Ca^2+^-Erk pathway during the negative selection of B cells [60]. In addition, PKCδ substantially impacts the survival and proliferation of mature follicular B cells. However, these studies did not provide direct evidence to indicate that PKCδ-regulated B cell tolerance in bone marrow and transitional B cells is required or sufficient for preventing lupus autoimmunity.

Moreover, to understand how the *PRKCD* G510S mutation contributes to juvenile-onset SLE (jSLE) in children [51], Moreews et al. introduced the *Prkcd* G510S mutation into the mouse genome [64]. They reported that *Prkcd^G510S/G510S^* mice had an early-onset severe autoimmune phenotype that included lymphoproliferation, kidney failure, various autoantibodies, a positive IFN score, and premature death. This phenotype closely resembled that observed in pediatric lupus patients carrying the *PRKCD^G510S/G510S^* mutation, identifying the *PRKCD^G510S/G510S^* mutation as a cause of jSLE. In addition, they showed that this lupus-like phenotype in *Prkcd^G510S/G510S^* mice was B cell-autonomous and that *Prkcd^G510S/G510S^* mutation altered the marginal zone (MZ), germinal center (GC), and plasma cells, suggesting the dysregulation of GC B cell tolerance in *Prkcd^G510S/G510S^* mice. Further mechanistic studies revealed that the mutated PKCδ protein was unresponsive to stimulation by the DAG analog PMA, demonstrating the loss of normal activity in the mutated PKCδ. However, autoimmune pathogenesis in *Prkcd^G510S/G510S^* mice appeared more severe than that in *Prkcd^−/−^* mice, suggesting that in addition to the loss of normal B cell tolerance function, the mutated PKCδ in *Prkcd^G510S/G510S^* mice acquired additional pro-autoimmune activities. Indeed, *Prkcd^G510S/G510S^* B cells have abnormally increased activity of the PI3K/mTOR pathway following BCR engagement, leading to lymphoproliferation. Treatment of *Prkcd^G510S/G510S^* mice with the mTORC1 inhibitor, rapamycin, partially attenuated disease activity, highlighting the detrimental effects of upregulated activation of the mTOR pathway in lupus pathogenesis. Given the critical role of mTOR in B cell selection and affinity maturation in the GC [65], this was not surprising, but a potential role for the mutated PKCδ in promoting the survival and development of lupus GC B cells in *Prkcd^G510S/G510S^* mice could not be ruled out. However, direct evidence supporting the regulation of GC B cell tolerance by PKCδ is still lacking. In addition, the status and importance of PKCδ activity in cases of lupus in the absence of *PRKCD* mutations are also unknown. Therefore, studying whether and how PKCδ regulates GC B cell tolerance, the key mechanism that prevents lupus autoimmunity, is indispensable for understanding PKCδ’s tolerance role in lupus pathogenesis.

## 5. PKCδ Regulates GC B Cell Tolerance

Our discovery that PKCδ in B cells is a critical enforcer of autoreactive B cell tolerance in the GC represents a major step forward in understanding the role of PKCδ in lupus autoimmunity [48]. We have shown that GC B cell-specific PKCδ-knockout mice exhibit a lupus-like phenotype including increased serum anti-dsDNA IgGs, proteinuria, and renal glomerular injury, indicating that GC B cell PKCδ is required for preventing lupus pathogenesis. The impaired GC B cell tolerance in these mice is evidenced by the increased survival of anti-dsDNA GC B cells, which is similar to what we observed in PKCδ-deficient mice, underscoring the requirement for PKCδ in deleting anti-dsDNA GC B cells.

**Figure 1:**
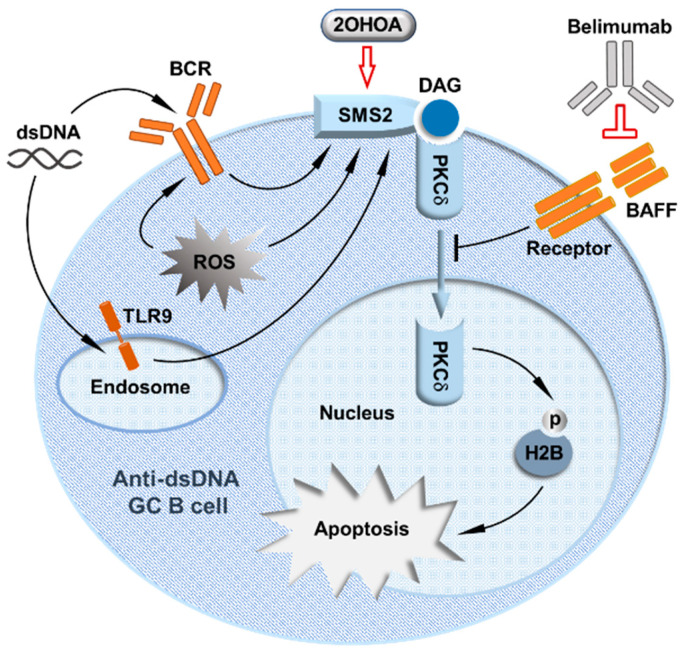
Model of a mechanism-oriented therapeutic approach to suppressing lupus autoimmunity by enhancing the SMS2/PKCδ-regulated germinal center B cell tolerance. This figure is adapted from Ref. [48], with modifications intended to highlight the new concept of lupus treatment that directly targets germinal center B cell tolerance.

Although lupus patients with *PRKCD* mutations are rare, our study of the regulation of PKCδ activity supports the idea that a deficit in PKCδ nuclear translocation in GC B cells is actually a common abnormality in lupus [48]. We discovered that PKCδ nuclear translocation in GC B cells is activated by SMS2, whose expression is drastically decreased in lupus patients’ B cells [48]. SMS2 forms a complex specifically with PKCδ to activate PKCδ nuclear translocation by SMS2-derived DAG. Although in normal mice, GC B cells express less PKCδ than naïve B cells, our further analysis of light zone (LZ) and dark zone (DZ) B cells showed that LZ B cells expressed significantly more *Prkcd* mRNA and PKCδ protein, coincident with significantly increased nuclear PKCδ in LZ B cells. On the other side, both *Sgms2* mRNA and SMS2 protein are highly upregulated in LZ B cells compared with DZ B cell and naïve B cells. Notably, anti-dsDNA GC B cells in the LZ have the highest SMS2 expression, coincident with SMS2-dependent PKCδ nuclear translocation, strongly supporting the more specific activation of PKCδ nuclear translocation by SMS2 in LZ anti-dsDNA GC B cells. Indeed, although SMS2 deficiency diminishes the nuclear PKCδ levels in GC B cells, these can be restored by DAG analog stimulation. SMS2 expression in cultured B cells can be upregulated by multiple signals, including by signals from the BCR, reactive oxygen species (ROS), and TLR9, which are all known to facilitate the negative selection of B cells in the GC. These data have demonstrated that SMS2 is the key regulator of PKCδ’s tolerance activity in the GC. Consistently, we found that SMS2-deficient mice exhibited a similar lupus-like phenotype with reduced apoptosis in anti-dsDNA GC B cells. Thus, we identified the SMS2/PKCδ pathway as a novel GC B cell tolerance mechanism that prevents lupus pathogenesis [48] (Figure 1), which broke new ground for understanding B cell tolerance in the GC. However, it is unclear why SMS2 expression in lupus patients’ B cells is generally reduced, and further studies are required to increase our understanding of this mechanism in the future.

## 6. PKCδ-Regulated GC B Cell Tolerance Is a Potential Target for Lupus Treatment 

The recognition of the key role of the SMS2/PKCδ pathway in preventing lupus pathogenesis also provided a new potential target to selectively suppress lupus autoimmunity by enhancing GC B cell tolerance. To test the concept of targeting the SMS2/PKCδ tolerance pathway for lupus treatment, we demonstrated that the SMS2/PKCδ tolerance pathway can be pharmacologically activated to relieve lupus pathogenesis in NZBWF1 mice, an established preclinical mouse model of lupus [48]. 2-hydroxyoleic acid (2OHOA), a small and safe molecular activator of SMS [66,67,68], was used to restore the attenuated SMS2/PKCδ tolerance pathway in NZBWF1 mice. We chose to activate SMS2 instead of directly targeting PKCδ nuclear translocation because there are no effective strategies to specifically activate PKCδ in GC B cells for two reasons: (1) the lack of specific activators of individual PKC isoforms [44], and (2) PKCδ levels are very low in GC B cells but much higher in naïve B cells [48]. In contrast, SMS2 is most highly expressed in anti-dsDNA GC B cells, and SMS2 specifically binds to PKCδ in GC B cells [48], providing a better target for specifically activating PKCδ nuclear translocation in GC B cells. In addition, 2OHOA has been proven to be a safe oral drug in a phase I/IIA clinical trial for glioma treatment [68]. We found that 2OHOA markedly relieved lupus pathogenesis in NZBWF1 mice, leading to a significant reduction in lupus-like kidney damage, proteinuria, and anti-dsDNA IgG titers. The SMS2/PKCδ tolerance pathway was required for 2OHOA’s effects, as evidenced by the finding that reductions in serum anti-dsDNA IgG titers were not observed in SMS2-deficient or PKCδ-deficient mice after treatment. Notably, the therapeutic effects of 2OHOA in NZBWF1 mice were associated with restored PKCδ tolerance activity in GC B cells without inhibiting total IgG production [48], strongly suggesting that targeting GC B cell tolerance is a feasible therapeutic approach that can selectively suppress lupus autoimmunity through deleting GC autoreactive B cells while sparing normal immune functions.

Moreover, the success of belimumab also points to the SMS2/PKCδ axis as a viable target for lupus therapy and justifies further exploration of how these pathways interact. A long-term clinical study reported that although belimumab treatment reduced the total numbers of both naïve B cells and activated B cells in lupus patients, the proportion of activated autoreactive B cells, but not naïve autoreactive B cells, was reduced [69]. The reduction in the proportion of activated autoreactive B cells caused by blockade of BAFF strongly suggests that, in addition to general B cell pro-survival activity, BAFF may counter GC B cell tolerance, possibly via its inhibition of PKCδ nuclear translocation (Figure 1). Notably, the SMS2/PKCδ pathway may also explain the modest efficacy of belimumab. We hypothesize that removing the BAFF-mediated inhibition of PKCδ nuclear translocation alone by belimumab may not be sufficient to restore PKCδ nuclear translocation without the adequate activation of PKCδ by SMS2-derived DAG. Therefore, as illustrated in Figure 1, the combination of belimumab and 2OHOA could be a more effective therapy for lupus. Further studies are needed to confirm belimumab’s effects on the SMS2/PKCδ tolerance pathway.

In summary, this review highlights the protective role of PKCδ against lupus autoimmunity by regulating B cell tolerance [48]. Our model demonstrates that in the GC, PKCδ nuclear translocation driven by SMS2-derived DAG is critical for deleting autoreactive anti-dsDNA GC B cells that arise as a result of SHM. The expression of B cell SMS2, a critical activator of PKCδ nuclear translocation, is markedly reduced in lupus patients’ B cells. Therefore, attenuated PKCδ-regulated apoptosis could be a key and shared pathophysiological feature that facilitates the emergence of lupus autoimmunity. The discovery of the SMS2/PKCδ pathway also offers the possibility of a viable and desirable therapeutic target that could enhance B cell tolerance in the GC without simultaneously suppressing protective antibody production. Such a therapeutic strategy could be more effective and have fewer adverse effects than current treatments for lupus. Further study of the SMS2/PKCδ tolerance pathway is warranted to vet this new therapeutic strategy, with the ultimate goal of improving patient outcomes.

## Figures and Tables

**Table 1:** Published mutations in *PKCRD* and *Prkcd* genes relevant to lupus autoimmunity. Relevant articles have been collected by a PubMed search using “*PKCRD*”, “*Prkcd*”, “mutation”, “SLE”, and “lupus” as keywords.

Reference	Human/Mouse	*PKCRD*/*Prkcd* Mutations	Relevant Findings
Salzer et al. [49], 2013	Human	Loss-of-function splice-site mutation within the catalytic domain of *PRKCD* c.1352 + 1G>A. No expression of PKCδ.	Severe autoimmunity with membranous glomerulonephritis, hepatosplenomegaly, and generalized lymphadenopathy. Positive for anti-nuclear antibodies and anti-dsDNA antibodies.
Kuehn et al. [50], 2013	Human	*PRKCD* c.1840C>T, p.Arg614Trp. Reduced PKCδ expression.	Autoimmunity with chronic lymphadenopathy, splenomegaly, autoantibodies, and elevated immunoglobulins, similar to the phenotype observed in PKCδ-deficient mice. Strongly positive for anti-nuclear antibodies, and negative for anti-dsDNA antibodies.
Belot et al. [51], 2013	Human	*PRKCD* c.1258G>A p.Gly510Ser. Reduced expression and activity of PKCδ.	Lupus autoimmunity with lupus nephritis. Patients have increased numbers of immature B cells in association with increased proliferation and decreased apoptosis. Positive for anti-nuclear antibodies and anti-dsDNA antibodies.
Kiykim et al. [52], 2015	Human	*PRKCD* c.742G>A p.Gly248Ser	Lupus-like disorder with erythematous skin rash. The patient has increased numbers of CD19^+^ B cells and naïve B cells. Positive for anti-nuclear antibodies, and negative for anti-dsDNA antibodies.
Nanthapisal et al. [53], 2017	Human	*PRKCD* c.1294G>T; p.Gly432Trp	Lupus autoimmunity with scarring alopecia, rash affecting the scalp, a photosensitive malar rash, and hepatosplenomegaly. Positive for anti-nuclear antibodies and anti-dsDNA antibodies.
Lei et al. [54], 2018	Human	*PRKCD* c.1294G > T; p.Gly432Trp	Lupus autoimmunity with acute cutaneous lupus, non-scarring alopecia, hemolytic anemia, and thrombocytopenia. Positive for anti-nuclear antibodies and anti-dsDNA antibodies.
Sharifinejad et al. [55], 2022	Human	*PRKCD c.1293_1294insA*	Autoimmunity with lymphoproliferation, recurrent pneumonia, cardiomyopathy, and dermatological manifestations.
Mecklenbrauke et al. [57], 2002	Mouse	Targeted disruption of *Prkcd* by replacing Exon1 with a LacZ/neo cassette. No expression of PKCδ.	A lupus-like autoimmune phenotype with splenomegaly and lymphadenopathy. The mice have increased numbers of B cells. Positive for anti-nuclear antibodies and anti-DNA antibodies.
Miyamoto et al. [58], 2002	Mouse	Targeted disruption of *Prkcd* by replacing Exon1 and Exon 2 with a neomycin-resistance cassette. No expression of PKCδ.	A lupus-like phenotype with glomerulonephritis. The mice have an expanded B cell population and the spontaneous formation of numerous GCs. Positive for anti-chromatin antibodies.
Limnander et al. [59], 2011	Mouse	Targeted disruption of *Prkcd* by replacing Exon1 with a LacZ/neo cassette. No expression of PKCδ [57].	Impaired activation of the proapoptotic Ca^2+^-ERK pathway during the negative selection of immature bone marrow B cells.
Limnander et al. [60], 2014	Mouse	Targeted disruption of *Prkcd* by replacing Exon1 with a LacZ/neo cassette. No expression of PKCδ [57].	Impaired antigen-dependent negative selection of splenic transitional B cells.
